# Risk Factors for Postoperative Atrial Fibrillation in Myocardial Revascularization Surgery: A 15-Year Experience

**DOI:** 10.3390/jcm13175171

**Published:** 2024-08-31

**Authors:** Diana Marcela Bonilla-Bonilla, Luis Miguel Osorio-Toro, Jorge Enrique Daza-Arana, Jhon H. Quintana-Ospina, Juan Carlos Ávila-Valencia, Heiler Lozada-Ramos

**Affiliations:** 1 Internal Medicine Specialization Program, Department of Health, Universidad Santiago de Cali, Santiago de Cali 760001, Colombia; diana.bonilla01@usc.edu.co (D.M.B.-B.); luis.osorio01@usc.edu.co (L.M.O.-T.); jhon.quintana00@usc.edu.co (J.H.Q.-O.); heiler@outlook.com (H.L.-R.); 2 Department of Research and Education, Clínica de Occidente SA, Santiago de Cali 760001, Colombia; rhcardiopulmonar09@gmail.com; 3 Genetics, Physiology, and Metabolism Research Group (GEFIME), Universidad Santiago de Cali, Santiago de Cali 760001, Colombia; 4 Health and Movement Research Group, Universidad Santiago de Cali, Santiago de Cali 760001, Colombia

**Keywords:** postoperative atrial fibrillation, myocardial revascularization surgery, risk factors

## Abstract

**Background:** Myocardial revascularization surgery (MRV) is a revascularization therapy for coronary artery disease aimed at improving survival conditions. Elderly patients with increased comorbidities undergoing MRV face challenges in preventing postoperative complications, including atrial fibrillation (AF), a common arrhythmia occurring in 40% of cases or even in 80% of cases if the procedure is combined with valve surgery. This study aimed to determine the risk factors associated with the appearance of postoperative AF (POAF) in patients undergoing isolated MRV. **Methods:** This is an epidemiological, retrospective, and analytical case–control study (90 cases and 360 controls). **Results:** Mortality within the group of patients who presented with POAF in the study population was 15.5%, and 9.44% in the control group. Logistic regression showed an association of AF with the presurgical variables age >60 years and urgent/emergency surgery and the postsurgical variables cardiogenic shock, blood transfusion, pulmonary edema, pleural effusion, orotracheal reintubation, and mechanical ventilation time. **Conclusions:** Strategies should be proposed for the timely identification of risk factors and postoperative complications related to AF onset to avoid the increased morbidity and mortality associated with this type of arrhythmia during the postoperative period.

## 1. Introduction

Coronary artery disease is one of the most common cardiovascular diseases worldwide, and coronary artery bypass grafting is the most frequently performed heart surgery in adults as part of treatment [1]. Postoperative atrial fibrillation (POAF) is the most common form of AF and the most common complication after heart surgery [2]. In the US, in 2017, of a total of 233,022 people undergoing CABG aortic and mitral valve surgeries (or combinations), 64,751 (27.8%) developed POAF [3].

The risk factors associated with the development of POAF—such as electrolyte imbalance, postoperative inotrope use, and absence of total myocardial revascularization surgery (MRV)—have been documented [4]. It has also been demonstrated that preoperative anxiety is a significant factor that increases the risk of developing this condition [5], along with older age, lower levels of creatinine clearance, and a larger diameter of the left atrium [6].

This clinical entity is very relevant because patients with POAF have longer hospital and intensive care unit (ICU) stays, higher hospital costs, and higher rates of short- and long-term adverse cardiovascular events [7]. It also significantly increases the risk of recurrent POAF [8,9]. In addition, this condition is an important factor in decreased 3-year survival [10]. In a recent systematic review and meta-analysis in patients undergoing coronary bypass, higher perioperative mortality, stroke, myocardial infarction, and acute kidney injury were documented. Likewise, these patients had a longer hospital stay and stay in intensive care, with higher mortality, stroke, and long-term atrial fibrillation [11], which evidences the finding of short- and long-term consequences after the presence of POAF.

This study aimed to determine the risk factors associated with the onset of POAF in patients undergoing isolated MRV in a high-complexity medical center in Santiago de Cali, Colombia.

## 2. Materials and Methods

### 2.1. Study Subjects and Design

A retrospective, case–control, analytical design was used to determine the factors associated with POAF in MRV surgery in a high-complexity medical center located in the city of Santiago de Cali, Colombia. This medical center is a regional reference setting for cardiovascular surgery care, with six ICUs for managing these patients.

The study included subjects 18 years of age or older at the time of the surgical intervention who had undergone isolated MRV surgery without another combined surgical intervention from 1 January 2006 to 31 December 2020. Subjects with missing data in their medical records, those with a previous diagnosis of AF, or those who had previously undergone heart surgery were excluded.

Cases were defined as adult patients undergoing isolated MRV surgery with AF evident in an electrocardiogram that was evaluated by a specialist within the first 7 days postsurgery. The information was organized in a master database. Then, a descriptive analysis of the risk factors was conducted in the general population, cases, and controls, taking into account the presurgical, trans-surgical, and postsurgical variables.

The sample size was calculated with a confidence level (CI) of 95%, α = 0.05, a statistical power of 80%, β = 0.2, a ratio of four controls for each case (1:4), and an adjustment of 10% of losses. The exposure values, according to a reference study by the European Society of Cardiology [12], were considered, which showed postsurgical kidney injury as a risk factor for POAF (case exposure: 17.6%; control exposure: 6.3%). This resulted in a sample where it was determined that, for a total size of 450 subjects, at least 90 cases and 360 controls were required. The cases and controls were selected randomly from the health institution’s database using the sample selection tool of the Epidat 4.2 software (Program for epidemiological data analysis. Ministry of Health, Xunta de Galicia, Spain; Pan American Health Organization (PAHO-WHO); CES University, Colombia), according to the inclusion and exclusion criteria.

Selection bias was controlled through randomization in the selection of the study sample and with the operational definition of case and control, obtaining the finding or diagnosis of atrial fibrillation from a reliable source for this purpose, which also reduces the misclassification bias. Information bias was controlled with the standardization of the measurement procedure as well as the training of personnel with the instrument and with data collection, which reduced the presence of measurement errors. Adjustment for confounding variables was included in the statistical analysis, which allowed adequate control of these. Regarding losses, this was anticipated in the study design by including the adjustment of the sample size with a percentage of overestimation.

The research was conducted in accordance with the international recommendations based on the Declaration of Helsinki [13,14]. The project was approved by the Technical Scientific Committee of the Clinical Institution and the Ethics and Bioethics Committee of the Department of Health of the Universidad Santiago de Cali (Act N° 21/2022).

### 2.2. Data Collection

The information was collected in the study retrospectively through a written and digital form completed by the researcher in the Epi Info™ 7.1.4 software, a program developed by the United States Centers for Disease Control and Prevention (CDC), which contained the variables under study. The information was obtained from the patients’ medical history documents. Additionally, when missing data were found, information was obtained from the postsurgical controls carried out by the institution’s cardiovascular surgeons.

To collect information, a pilot test was carried out by the researchers on a group of patients with characteristics similar to that of the study population, which allowed the strengthening of learning in the application of the instrument.

The consolidation of information was carried out in a master database that allowed the consolidation of the variables contemplated in the data collection instrument after evaluating the agreement and statistical validation of the databases to avoid systematic errors in typing and guarantee the quality of the data. The purification of the information was carried out by creating frequency distributions and simple tables in each of the variables with the objective of identifying incorrect codes and inconsistent information, which was verified and corrected as appropriate with the objective of guaranteed reliability in the study.

### 2.3. Statistical Analysis

A descriptive analysis of the variables was initially conducted in the general population, cases, and controls. This description was developed for categorical data through frequency distribution, relative frequencies, and proportions. A numerical analysis of measures of central tendency and dispersion was performed for the quantitative data, and the Student’s *t*-test was used to test the hypotheses of differences between sample means. To apply this test, the statistical assumption of normality (Kolmogorov–Smirnov test) and homogeneity in variance (Levene test) of the continuous data were additionally taken into account, which were obtained from a random sample of the study population.

It was determined whether the exposure variables had an independent effect on the likelihood of case occurrence with the corresponding 95% CI. Then, using a chi-squared test, the bivariate associations between the cases and each of the exposures of interest were estimated to evaluate whether there were statistically significant differences between the prevalence values of exposures to qualitative variables. For the chi-square test, the statistical assumption was that at least 80% of the expected frequencies were greater than 5.

The variables for the construction of the final model were identified using statistical criteria with bivariate associations and according to the risk factors reported in the scientific evidence. The strength of the association was measured using the odds ratio (OR) with a significance level of 0.05 and a CI of 95%. The criteria for assigning the nonexposure category were based on the literature review of the factors associated with the event under study and/or on the categories of the variables with less frequency in cases of AF. A multivariate analysis was then performed, which included a logistic regression model. The explanatory (independent) variables with *p* ≤ 0.25 in the univariate models were introduced into the initial logistic model. According to statistical criteria, the final explanatory model was derived using a step-by-step retrograde elimination procedure (forward to back), with an entry likelihood of <0.10 and a withdrawal likelihood of >0.25, including the statistically significant factors that contribute to the explanation of the variability of the event (Pseudo R^2^). In this step, the collinearity of the factors was evaluated, and those with high collinearity were excluded.

Confounding, effect modification, and interaction tests were performed between variables of the adjusted model. The model was validated using the Hosmer–Lemeshow goodness-of-fit test and the likelihood ratio. The data analysis and processing were conducted using the STATA 17.0^®^ statistical software (StataCorp, College Station, TX, USA).

## 3. Results

A total of 450 patients were included for case–control analysis (cases *n* = 90, controls *n* = 360) during the study period. The average age was 69.6 ± 7.7 years for the case group and 65.06 ± 9.3 years for the control group, with a general average of 66.0 ± 9.2 years. In both groups, most of the study population consisted of men (cases, 67.8%; controls, 72.5%). Regarding personal history, arterial hypertension was similar in the two groups analyzed, and it was reported as a pathological history in 90% of cases and 89.7% of controls.

When comparing the frequencies of exposure to the presurgical variables (Table 1) in the cases, the following was evident: a tendency toward older age, a history of chronic obstructive pulmonary disease, peripheral vascular disease, presurgical hospital stay longer than 8–14 days, urgent/emergency surgery, lower body mass index, and histories of moderate mitral regurgitation and mitral stenosis with *p* < 0.05, considering the differences between the groups as statistically significant. Severe valve insufficiencies were minimal because this clinical condition is associated with combined MRV surgery and valve replacement, which were excluded from this study.

Among the exposure factors during the surgical procedure, longer extracorporeal circulation time with differences in the average values of pump time and ischemia (*p* < 0.05) was determined in individuals who developed AF, without statistically significant differences in the remaining variables (Table 2).

In the postsurgical intervention period (Table 3), the cases had a longer stay in the ICU; a higher frequency of blood transfusion (especially in the category of >3 units of red blood cells and >2 units of plasma); a higher frequency of hemodynamic support provided by the use of vasopressors, inotropics, and vasodilators; a higher frequency of cardiogenic shock; venous oxygen saturation (SvO_2_) < 60%; and a longer mechanical ventilation time (*p* < 0.05).

Complications occurred at a higher percentage in patients with POAF, which included pulmonary edema, acute respiratory distress syndrome, pleural effusion requiring drainage, tracheal intubation, ischemic stroke, delirium, sepsis, surgical site infection, acute kidney failure with dialysis, and coagulopathy (*p* < 0.05). Among the characteristics of the day of occurrence of AF (Table 4), the average time of presentation of arrhythmia was 2.3 days. In terms of electrolytes, hypocalcemia and hypermagnesemia were characterized by average values of 1.1 mmol/L and 2.8 mg/dL, respectively. Mortality from POAF in the study population was 15.5% in the cases and 9.4% in the controls. Cardiogenic shock was the cause of death with the highest percentage, followed by sepsis (Table 5).

Different logistic models were conducted according to the theoretical working model and the statistical criteria to identify the variables whose statistical significance and theoretical importance allowed for the construction of the model.

Collinearity was analyzed before constructing the final model using the multicollinearity index, considering it positive if the variance inflation factor (VIF) was >5 or the correlation coefficient was ≥0.90. Collinearity was found between postsurgical cardiogenic shock and the SvO_2_ variable; the latter was thus excluded from the regression model. For the age variable, an analysis was performed with different cut-off points; it was found that being 60 years or older was the age with the best prediction of POAF, so it was included in the logistic model with this category.

Eight variables were included in the final logistic model (Table 6). Presurgical variables were age ≥60 years and urgent/emergency surgery, whereas the postsurgical variables were cardiogenic shock, blood transfusion, pulmonary edema, pleural effusion, tracheal reintubation, and mechanical ventilation time. The resulting model demonstrated 26% variability in AF events after MRV (Pseudo R^2^).

**Figure 1:**
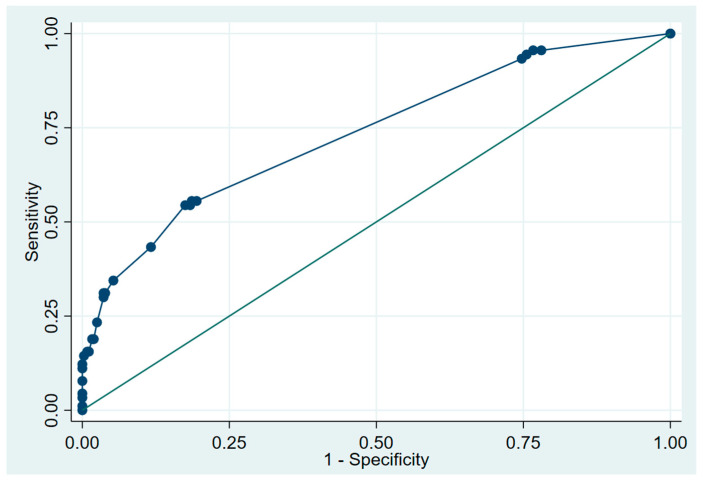
ROC curve and AUC of the POAF prediction regression model.

The evaluation of the fit of the final logistic regression model showed a good fit to the data (Hosmer–Lemeshow) with a chi-squared value of 15.28 (*p* = 0.760). Furthermore, the model demonstrated a capacity to classify cases as cases with 26% (sensitivity) and the capacity to classify controls as controls with 97% (specificity). That is, the model has a high capacity for ruling out patients unlikely to have AF. The probability of being a case among those predicted by the model was 68%, whereas the probability of being a control among patients without AF, according to the model, was 84%. Furthermore, the AUC of the developed model was 0.75, showing good discrimination (Figure 1).

## 4. Discussion

This study was conducted with a population undergoing isolated MRV surgery in a third-level clinic and reference center in cardiovascular surgery in southwestern Colombia and sought to determine the risk factors associated with the appearance of POAF among patients undergoing isolated MRV. A descriptive analysis of the variables was initially conducted in the general population and in cases and controls over a 15-year period, including 450 patients.

The adjusted ORs in the final logistic model with a statistically significant association were age ≥60 years, urgent or emergency surgery, postsurgical cardiogenic shock, postsurgical blood transfusion, postsurgical pulmonary edema, postsurgical pleural effusion, tracheal reintubation, and mechanical ventilation time >2 days.

New-onset POAF is frequent, and rates of up to 60% have been described [15]. Hospital stay is a factor most frequently associated with POAF. Sotiris et al. reported that POAF is independently associated with length of hospital stay, with an average of 9 days (*p* < 0.001) [16]. This finding is similar to that of our study, which found a higher percentage in cases with a hospital stay of >2 days (91.1% vs. 80.3% *p* = 0.016).

Age is one of the factors most frequently associated with the onset of POAF in patients undergoing MRV, with a cut-off age >75 years [17]. Similarly, Almassi et al. reported a 1.6-fold increase in the incidence of POAF for each additional decade of life [18], and Villareal et al. determined that age over 65 years is an independent risk factor for developing POAF after revascularization (OR, 2.4; 95% CI, 2.06–2.74; *p* < 0.0001) [19]. A greater rate of POAF was observed in the age range between 55 and 74 years in this study, and an adjusted OR of 4.1 (*p* = 0.001) for age ≥60 years was observed in the logistic model. However, the study by Cureus et al. showed that age >65 years was associated with the appearance of AF but was not statistically significant (*p* > 0.05) [20].

Hemodynamic status has also been associated with the incidence of POAF, and our study estimated a greater likelihood of exposure to cardiogenic shock in this event (OR, 2.5, 95% CI: 1.28–4.77, *p* = 0.007). Hakala et al. constructed a multivariate logistic regression model to determine risk factors for POAF after MRV and reported that increasing age (*p* < 0.001), preoperative use of digoxin (*p* = 0.003), the need for an intra-aortic balloon pump, inotropic medication when weaning from cardiopulmonary bypass during the first 24 h after surgery (*p* = 0.013), the increase in surface area (*p* = 0.006), and a lower ejection fraction (*p* = 0.048) were independent risk factors for POAF [21].

Another finding related to the hemodynamic context that was obtained in this study was the statistically significant difference in moderate mitral regurgitation and mitral stenosis between cases and controls. This relationship is important because it explains how the increase in volume or pressure overload leads to remodeling at the left atrium level [22]. Furthermore, the postoperative use of inotropes has been associated with this entity [23]. The use of vasodilators and vasopressors has also been described as risk factors for POAF [24,25], as shown in this case–control investigation.

In our study, the highest OR (5.4) was obtained for postsurgical pulmonary edema (95% CI, 2.26–12.84; *p* = 0.030), which suggests a high probability of occurrence of POAF with this risk factor, considering that no collinearity was observed in statistical analysis. Despite limited evidence of this relationship in patients after myocardial revascularization, acute lung edema is noted as an emergency requiring prompt treatment in cardiovascular disease cases and is considered a triggering factor for AF [26].

Conclusively, three findings were identified in this investigation, which were scarcely described in the literature on patients with POAF: postsurgical pleural effusion (OR, 3.0; 95% CI, 1.57–5.54; *p* = 0.001), tracheal reintubation (OR, 3.1; 95% CI, 1.24–7.51; *p* = 0.015), and time of invasive mechanical ventilation >2 days (OR, 1.2; 95% CI, 1.07–2.31; *p* = 0.025). They represent clinical conditions susceptible to timely intervention in the postoperative period of the ICU. Experimental evidence suggests that pleural effusion can mimic a clinical picture similar to cardiac tamponade [27], to the extent that some authors call it “thoracic tamponade” [28]. Sadaniantz et al. revealed that 18% of patients with pleural effusion exhibited echocardiographic signs of right atrium collapse and increased pressure in the pulmonary artery [29]. This explains the causal relationship with AF, emphasizing the need for active detection and timely intervention. Likewise, a previous study reported a higher frequency of AF among patients with prolonged mechanical ventilation (31% vs. 11.3%, *p* < 0.001) [30].

Patients with blood transfusions have a higher risk of POAF, with an OR of 1.6 (*p* = 0.030). This association is greater with the transfusion of blood products from red blood cells and plasma, a finding that was also demonstrated in a meta-analysis of cohort studies [31]. Furthermore, the scientific literature has reported a relationship between increased bleeding, transfusion requirements, and a higher risk of AF incidence in the postsurgical period for patients with MRV [32].

Patients undergoing nonurgent cardiovascular surgical procedures who can be taken to prehabilitation obtain great benefits and a decrease in postsurgical complications [33]. This is contrary to what this study found, where urgent or emergency surgery (OR, 2.1; 95% CI 1.06–4.85, *p* = 0.044) presented a higher risk of presenting POAF, a clinical finding rarely described in the literature. The context of patients undergoing urgent or emergency cardiovascular surgery is associated with higher mortality rates and hospital complications [34].

Immune cells and proteins that mediate the inflammatory response in cardiac tissue and circulatory processes are associated with AF. Furthermore, the presence of inflammation in the heart or systemic circulation can predict the onset of AF and recurrence in the general population, as well as in patients after cardiac surgery [35]. This is where extracorporeal circulation and cardiogenic shocks will not be medical conditions unrelated to this condition.

Mortality among patients with POAF is well documented [9]. With respect to mortality and cause of death in the case group, our study demonstrated in the group of cases that the median occurred at 6 days, the causes being cardiogenic shock in 57.2%, followed by sepsis in 42.8%. Conversely, the control group showed a median of less than 2 days, with similar causes, including bleeding and acute myocardial infarction. In our study, mortality was higher in the POAF group at 15.5% than in the control group at 9.44%, a trend similar to that demonstrated in the study by Kaw et al. [36].

### 4.1. Clinical Implications of the Study

The factors reported in this study have significant clinical implications in addition to the statistical significance found in predicting POAF. Developing strategies to identify risk factors and provide timely treatment for the identified variables is crucial to reducing the onset of POAF and the associated complications, exerting a strong impact on the well-being of patients and the health system and, therefore, controlling the factors. Modifiable elements of the predictive model developed in this study favor the prognosis of this population.

### 4.2. Study Limitations

This study included a small number of patients who experienced new-onset AF after MRV. While this provides a trend, ensuring data reliability necessitates adequate study power. A larger study, possibly a multicenter one, is needed to effectively cover all aspects of AF following coronary artery bypass grafting. Larger prospective studies are required to validate scores as a stratification tool for the likelihood of spontaneous AF conversion [37].

## 5. Conclusions

In this case–control study of patients with POAF after MRV, it was determined that POAF is a very common and potentially fatal complication. Conditions such as elderly age, undergoing urgent or emergency surgery, cardiogenic shock, blood transfusion, pulmonary edema, pleural effusion, tracheal reintubation, and longer mechanical ventilation time were significantly associated with POAF. Strategies should be established for risk factor identification and timely treatment of these variables in order to reduce the occurrence of POAF and the complications associated with it. Likewise, multicenter studies should be conducted to validate the reported data.

## Figures and Tables

**Table 1:** Description of presurgical variables.

Variable	Cases AF *n* = 90	Controls No AF *n* = 360	Total *n* = 450	*p*-Value
	*n* (%)	*n* (%)	*n* (%)	
Age (years)				0.001
<55	6 (6.7)	43 (11.9)	49 (10.9)	
55–64	16 (17.8)	130 (36.2)	146 (32.4)	
65–74	43 (47.8)	120 (33.3)	163 (36.2)	
≥75	25 (27.8)	67 (18.6)	92 (20.4)	
Average ± SD ^1^	69.6 ± 7.7	65.1 ± 9.3	66.0 ± 9.2	0.000
BMI (kg/m^2^)				0.007
<25	48 (53.3)	136 (37.8)	184 (40.9)	
25–29	32 (35.6)	178 (49.4)	210 (46.7)	
30–34	6 (6.7)	38 (10.6)	44 (9.8)	
≥35	4 (4.4)	8 (2.2)	12 (2.7)	
Average ± SD ^1^	25.3 ± 4.1	26.0 ± 3.6	25.9 ± 3.7	0.106
Body surface (m^2^)				0.420
<1.5	6 (6.7)	21 (5.8)	27 (6.0)	
1.5–1.74	38 (42.2)	137 (38.2)	175 (38.9)	
1.75–1.99	37 (41.1)	165 (45.8)	202 (44.9)	
≥2.0	9 (10)	37 (10.3)	46 (10.2)	
Average ± SD ^1^	1.8 ± 0.2	1.8 ± 0.2	1.8 ± 0.2	0.349
Sex				
Female	29 (32.2)	99 (27.5)	128 (28.4)	
Male	61 (67.8)	261 (72.5)	322 (71.6)	0.374
Socioeconomic				
Stratum				
High	4 (4.4)	32 (8.9)	36 (8.0)	
Middle	60 (66.7)	222 (61.7)	282 (62.7)	0.164
Low	26 (28.9)	106(29.4)	132 (29.3)	
Smoker				
No	42 (46.7)	168 (46.7)	210 (46.7)	
Yes ^2^	48 (53.3)	192 (53.3)	240 (53.3)	1.00
Diabetes mellitus				
No	52 (57.8)	218 (60.6)	270 (60.0)	
Insulin-dependent	14 (15.6)	61 (16.9)	75 (16.7)	0.403
Noninsulin-dependent	24 (26.7)	81 (22.5)	105 (23.3)	
Dyslipidemia				
No	36 (40)	144 (40)	180 (40)	
Yes	54 (60)	216 (60)	270 (60)	1.00
Heart Failure				
No	61 (67.8)	279 (77.5)	340 (75.6)	
Yes	29 (32.2)	81 (22.5)	110 (24.4)	0.055
LVEF				
≥35%	79 (87.8)	332 (92.2)	411 (91.3)	0.180
<35%	11 (12.2)	28 (7.8)	39 (8.7)	
Average ± SD ^1^	50.7 ± 10.9	51.8 ± 9.9	51.5 ± 10.1	0.385
Arterial hypertension				
No	9 (10)	37 (10.3)	46 (10.2)	
Yes	81 (90)	323 (89.7)	404 (89.8)	0.938
COPD:				
No	70 (77.8)	321(89.2)	391 (86.9)	0.004
Yes	20 (22.2)	39(10.8)	59 (13.1)	
Sepsis				
No	81 (90)	335 (93.1)	416 (92.4)	0.327
Yes	9 (10)	25 (6.9)	34 (7.6)	
Chronic kidney failure				
No	70 (77.8)	318 (88.3)	388 (86.2)	
Without dialysis	15 (16.7)	32 (8.9)	47 (10.4)	0.009
With dialysis	5 (5.6)	10 (2.8)	15 (3.3)	
Renal function				0.199
Creatinine level: <2.3 mg/dL	83 (92.2)	344 (95.6)	427 (94.9)	
Creatinine level: ≥2.3 mg/dL	7 (7.8)	16 (4.4)	23 (5.1)	
Average ± SD ^1^	1.3 ± 1.3	1.2 ± 1.1	1.2 ± 1.2	0.338
Peripheral vascular disease				
No	73 (81.1)	324 (90)	397 (88.2)	0.019
Yes	17 (18.9)	36 (10)	53 (11.8)	
Pulmonary hypertension				
No	70 (77.8)	310 (86.1)	380 (84.4)	
Mild-Moderate	16 (17.8)	43 (11.9)	59 (13.1)	0.051
Severe	4 (4.4)	7 (1.9)	11 (2.4)	
Stroke				
No	85 (94.4)	349 (96.9)	434 (96.5)	0.252
Yes	5 (5.6)	11 (3.1)	16 (3.6)	
Arrhythmia				
No	86 (95.6)	346 (96.1)	432 (96.0)	0.810
Yes	4 (4.4)	14 (3.9)	18 (4.0)	
Type of arrhythmia				
Atrial fibrillation	0 (0)	0 (0)	0 (0)	-
Supraventricular tachycardia	0 (0)	6 (1.7)	6 (1.3)	-
Ventricular tachycardia	0 (0)	2 (0.6)	2 (0.4)	-
Ventricular fibrillation	0 (0)	2 (0.6)	2 (0.4)	-
Extrasystole	2 (2.2)	5 (1.4)	7 (1.6)	0.568
Other	2 (2.2)	0 (0)	2 (0.4)	-
AMI				
No	52 (57.8)	193 (53.6)	245 (54.4)	
>21 days	14 (15.6)	57 (15.8)	71 (15.8)	0.478
8–21 days	17 (18.9)	80 (22.2)	97 (21.6)	
≤7 days	7 (7.8)	30 (8.3)	37 (8.2)	
Previous percutaneous intervention				
No	66 (73.3)	287 (79.7)	353 (78.4)	0.187
Yes	24 (26.7)	73 (20.3)	97 (21.6)	
Use of IABP				
No	86 (95.6)	351 (97.5)	437 (91.2)	0.325
Yes	4 (4.4)	9 (2.5)	13 (2.9)	
Angina:				
No	46 (51.1)	211 (58.6)	257 (57.1)	0.198
Yes	44 (48.9)	149 (41.4)	193 (42.9)	
Hospital stay				
≤7 days	56 (62.2)	261 (72.5)	317 (70.4)	
8–14 days	31 (34.4)	83 (23.1)	114 (25.3)	0.026
≥15 days	3 (3.3)	16 (4.4)	19 (4.2)	
Average ± SD ^1^	6.2 ± 4.9	5.9 ± 5.0	6.0 ± 4.9	0.540
Number of injured vessels				
One vessel	1 (1.1)	23 (6.4)	24 (5.3)	
Two vessels	16 (17.8)	45 (12.5)	61 (13.6)	0.046
≥Three vessels	73 (81.1)	292 (81.1)	365 (81.1)	
Left main coronary artery lesion				
No	65 (72.2)	290 (80.6)	355 (78.9)	0.083
Yes	25 (27.8)	70 (19.4)	95 (21.1)	
Hemoglobin:				
≥12 g/dL	58 (64.4)	266 (73.9)	324 (72)	0.074
<12 g/dL	32 (35.6)	94 (26.2)	126 (28)	
Average ± SD ^1^	12.7 ± 1.9	13.1 ± 1.8	13.1 ± 1.8	0.057
Blood transfusion: ^3^				
No	87 (96.7)	351 (97.5)	438 (97.3)	0.661
Yes	3 (3.3)	9 (2.5)	12 (2.7)	
Type of surgery				
Elective	76 (84.4)	338 (93.9)	36 (8)	0.003
Urgent/Emergency	14 (15.6)	22 (6.1)	414 (92)	
Use of vasodilators				
No	70 (77.8)	295 (81.9)	365 (81.1)	0.366
Yes	20 (22.2)	65 (18.1)	85 (18.9)	
Use of vasopressors				
No	89 (98.9)	356 (98.9)	445 (98.9)	1.00
Yes	1 (1.1)	4 (1.1)	5 (1.1)	
Use of inotropics				
No	89 (98.9)	355 (98.6)	444 (98.7)	0.837
Yes	1 (1.1)	5 (1.4)	6 (1.3)	
Ventilatory support:				
No	84 (93.3)	348 (96.7)	432 (96.0)	0.149
Yes	6 (6.7)	12 (3.3)	18 (4.0)	
Valve insufficiency				
Aortic				
Mild	12 (13.3)	30 (8.3)	42 (9.3)	0.145
Moderate	3 (3.3)	6 (1.7)	9 (2.0)	
Tricuspid				
Mild	13 (14.4)	39 (10.8)	52 (11.6)	
Moderate	0 (0)	5 (1.4)	5 (1.1)	0.338
Severe	1 (1.1)	0 (0.)	1 (0.2)	
Mitral				
Mild	26 (28.9)	99 (27.5)	125 (27.8)	
Moderate	11 (12.2)	14 (3.9)	25 (5.6)	0.002
Severe	1 (1.1)	0 (0)	1 (0.2)	
Valvular stenosis				
Aortic				
Yes	3 (3.3)	7 (1.9)	10 (2.2)	0.424
No	87 (96.7)	353 (98.1)	440 (97.8)	
Mitral				
Yes	2 (2.2)	1 (0.3)	3 (0.7)	0.043
No	88 (97.8)	359 (99.7)	447 (99.3)	

BMI: body mass index. LVEF: left ventricular ejection fraction. COPD: chronic obstructive pulmonary disease. AMI: acute myocardial infarction. IABP: intra-aortic balloon pump. ^1^ Standard deviation. ^2^ Current smoker or former smoker. ^3^ Red blood cells, plasma, platelets, or cryoprecipitate.

**Table 2:** Description of trans-surgical variables.

Variable	Cases AF *n* = 90	Controls No AF *n* = 360	Total *n* = 450	*p*-Value
	*n* (%)	*n* (%)	*n* (%)	
Revascularized vessels				
<3 vessels	12 (13.3)	61 (16.9)	73 (16.2)	0.406
≥3 vessels	78 (86.7)	299 (83.1)	377 (83.8)	
Extracorporeal circulation				
No	1 (1.1)	13 (3.6)	14 (3.1)	
Yes	89 (98.9)	347 (96.4)	436 (96.9)	0.222
Pump time				
≤90 min	72 (80)	313 (86.9)	385 (85.6)	0.094
>90 min	18 (20)	47 (13.1)	65 (14.4)	
Average ± SD ^1^	76.9 ± 19.9	70.7 ± 23.1	71.9 ± 22.6	0.020
Ischemia time				
<60 min	73 (81.1)	314 (87.2)	387 (86.0)	0.135
≥60 min	17 (18.9)	46 (12.8)	63 (14.0)	
Average ± SD ^1^	48.3 ± 14.2	44.3 ± 14.5	45.1 ± 14.5	0.020
Blood transfusion ^2^				
No	29 (32.2)	148 (41.1)	177 (39.3)	
Yes	61 (67.8)	212 (58.9)	273 (60.7)	0.123
Type of blood products				
Red blood cells				
≤3 units	48 (53.3)	171 (47.5)	219 (48.7)	0.322
>3 units	11 (12.2)	30 (8.3)	41 (9.1)	
Plasma				
≤2 units	29 (32.2)	109 (30.3)	138 (30.7)	0.720
>2 units	22 (24.4)	73 (20.3)	95 (21.1)	
Platelets				
≤6 units	8 (8.9)	18 (5)	26 (5.8)	0.157
>6 units	5 (5.6)	16 (4.4)	21 (4.7)	
Use of IABP				
No	80 (88.9)	336 (93.3)	416 (92.4)	0.154
Yes	10 (11.1)	24 (6.7)	34 (7.6)	
Cardiogenic shock				
No	83 (92.2)	344 (95.6)	427 (94.9)	0.199
Yes	7 (7.8)	16 (4.4)	23 (5.1)	
Arrhythmia				
No	87 (96.7)	342 (95)	429 (95.3)	0.503
Yes	3 (3.3)	18 (5)	21 (4.7)	
Type of arrhythmia				
Atrial fibrillation	0 (0)	0 (0)	0 (0)	
Supraventricular tachycardia	0 (0)	1 (0.3)	1 (0.2)	
Ventricular tachycardia	1 (1.1)	3 (0.8)	4 (0.9)	0.802
Ventricular fibrillation	0 (0)	8 (2.2)	8 (1.8)	
Other	1 (1.1)	0 (0)	1 (0.2)	
Use of vasopressors				
No	71 (78.)	284 (78.9)	355 (78.9)	1.00
Yes	19 (21.1)	76 (21.1)	95 (21.1)	
Use of inotropics				
No	43 (47.8)	187 (51.9)	230 (51.1)	0.479
Yes	47 (52.2)	173 (48.1)	220 (48.9)	
Use of vasodilators				
No	61 (67.8)	240 (66.7)	301 (66.9)	0.841
Yes	29 (32.2)	120 (33.3)	149 (33.1)	

IABP: intra-aortic balloon pump. ^1^ Standard deviation. ^2^ Red blood cells, plasma, platelets, or cryoprecipitate.

**Table 3:** Description of postsurgical variables.

Variable	Cases AF *n* = 90	Controls No AF *n* = 360	Total	*p*-Value
	*n* (%)	*n* (%)	*n* (%)	
Intensive care stay				
≤2 days	8 (8.9)	71 (19.7)	79 (17.6)	
>2 days	82 (91.1)	289 (80.3)	371 (82.4)	0.016
Hospital stay				
Not admitted to hospital	16 (17.8)	31 (8.6)	47 (10.4)	
Admitted to hospital	74 (82.2)	329 (91.4)	403 (89.6)	0.011
Blood transfusion ^2^				
No	38 (42.2)	237 (65.8)	275 (61.1)	
Yes	52 (57.8)	123 (34.2)	175 (38.9)	0.000
Type of blood products				
Red blood cells				
≤3 units	32 (35.6)	84 (23.3)	116 (25.8)	
>3 units	15 (16.7)	27 (7.5)	42 (9.3)	0.007
Plasma				
≤2 units	4 (4.4)	4 (1.1)	8 (1.8)	0.032
>2 units	16 (17.8)	41 (11.4)	57 (12.7)	
Platelets				
≤6 units	5 (5.6)	12 (3.3)	17 (3.8)	0.323
>6 units	2 (2.2)	5 (1.4)	7 (1.6)	
Cryoprecipitate				
<10 units	2 (2.2)	9 (2.5)	11 (2.4)	0.879
≥10 units	2 (2.2)	2 (0.6)	4 (0.9)	
Use of vasopressors				
No	50 (55.6)	260 (72.2)	310 (68.9)	
Yes	40 (44.4)	100 (27.8)	140 (31.1)	0.002
Use of inotropics				
No	22 (24.4)	150 (41.7)	172 (38.2)	
Yes	68 (75.6)	210 (58.3)	278 (61.8)	0.003
Use of vasodilators				
No	39 (43.3)	200 (55.6)	239 (53.1)	
Yes	51 (56.7)	160 (44.4)	211 (46.9)	0.038
Use of IABP				
No	80 (88.9)	335 (93.1)	415 (92.2)	
Yes	10 (11.1)	25 (6.9)	35 (7.8)	0.187
Cardiogenic shock				
No	66 (73.3)	326 (90.6)	392 (87.1)	
Yes	24 (26.7)	34 (9.4)	58 (12.9)	0.000
SvO_2_				
≥60%	68 (75.6)	315 (87.5)	383 (85.1)	0.004
<60%	22 (24.4)	45 (12.5)	67 (14.9)	
Average ± SD ^1^	65.4 ± 10.1	68.8 ± 8.3	68.2 ± 8.8	0.001
Invasive mechanical ventilation time				
≤2 days	56 (62.2)	310 (86.1)	366 (81.3)	
>2 days	34 (37.8)	50 (13.9)	84 (18.7)	0.000
Cardiorespiratory Complications				
Pulmonary edema	18 (20)	12 (3.3)	30 (6.7)	0.000
AMI	1 (1.1)	3 (0.8)	4 (0.9)	0.802
ARDS	3 (3.3)	1 (0.3)	4 (0.9)	0.006
Hemothorax	4 (4.4)	5 (1,4)	9 (2)	0.064
Pneumothorax	3 (3.3)	0 (0)	3 (0.7)	-
Pleural effusion requiring drainage	29 (32.2)	36 (10)	65 (14.4)	0.000
Tracheal reintubation	15 (16.7)	15 (4.2)	30 (6.7)	0.000
Tracheostomy	4 (4.4)	5 (1.4)	9 (2)	0.064
Postsurgical bleeding	14 (15.6)	34 (9.4)	48 (10.7)	0.093
VAP	3 (3.3)	7 (1.9)	10 (2.2)	0.424
Surgical reintervention	18 (20)	45 (12.5)	63 (14)	0.067
Atelectasis	16 (17.8)	45 (12.5)	61 (13.6)	0.191
Neurological complications				
Ischemic stroke	6 (6.7)	1 (0.3)	7 (1.6)	0.000
Delirium	18 (20)	32 (8.9)	50 (11.1)	0.003
TIA	0 (0)	1 (0.3)	1 (0.2)	-
Gastrointestinal complications				
Intestinal bleeding	4 (4.4)	5 (1.4)	9 (2)	0.064
Infectious complications				
Sepsis	26 (28.9)	54 (15)	80 (17.8)	0.002
Mediastinitis	7 (7.8)	13 (3,6)	20 (4.4)	0.086
Surgical site infection	15 (16.7)	33 (9.2)	48 (10.7)	0.039
Kidney complications				
Kidney failure without dialysis	8 (8.9)	15 (4.2)	23 (5.1)	
Kidney failure with dialysis	10 (11.1)	8 (2.2)	18 (4)	0.000
Hematological complications:				
Coagulopathy	10 (11.1)	9 (2.5)	9 (4.2)	0.000

IABP: intra-aortic balloon pump. AMI: acute myocardial infarction. ARDS: acute respiratory distress syndrome. VAP: ventilator-associated pneumonia. TIA: transient ischemic attack. SvO_2_: Venous oxygen saturation. ^1^ Standard deviation. ^2^ Red blood cells, plasma, platelets, or cryoprecipitate.

**Table 4:** Characteristics of the day of atrial fibrillation occurrence (*n* = 90).

Variable	Sodium (mEq/L)	Potassium (mEq/L)	Chlorine (mEq/L)	Magnesium (mg/dL)	Calcium (mg/dL)	Day of AF
Average	140.2	4.2	105.8	2.8	1.1	2.3
Standard deviation	4.8	0.8	5.1	0.6	0.3	1.5
Q1 (25%)	137.2	3.7	102.0	2.4	1.0	1.0
Q2 (50%)	140.0	4.1	106.0	2.9	1.0	2.0
Q3 (75%)	143.0	4.4	109.0	3.2	1.1	3.0
Maximum	150.0	6.9	117.0	3.8	3.0	7.0
Minimum	128.0	2.6	93.3	1.5	0.8	1.0

Place of occurrence: ICU: 100%. AF resolution: Pharmacological: 100%. Anticoagulation required: Yes: 11.1%.

**Table 5:** Day and cause of death of cases and controls.

Day and Cause of Death in Cases (*n* = 14)							
Day of Death	*n*	%	Cause of Death	*n*	%	Average ± SD ^1^	Median
≤2 days	6	42.8%	Sepsis	6	42.8%	19.5 days ± 10.8	20 days
>2 days	8	57.2%	Cardiogenic shock				
Average: 10.7 days ± 10.7 ^1^				8	57.2%	4.1 days ± 10.7	2 days
Median: 6 days							
**Day and Cause of Death in Controls (*n* = 34)**							
**Day of death**	***n***	**%**	**Cause of death**	***n***	**%**	**Average ± SD ^1^**	**Median**
≤2 days	23	67%	Sepsis	7	20.6%	22.4 days ± 9.9	30 days
>2 days	11	33%	Bleeding	6	17.7%	1.33 days ± 9.1	1 day
Average: 6 days ± 9.9 ^1^			Cardiogenic shock	20	58.8%	1.65 days ± 8.6	1 day
Median: 2 days			AMI	1	2.9%	9 days ± 0.0	9 days

AMI: acute myocardial infarction. ^1^ Standard deviation.

**Table 6:** Adjusted odds ratios of the final logistic model.

Variable	Adjusted OR	95% CI	*p*-Value
Age ≥60 years	4.1	1.82–9.36	0.001
Urgent/emergency surgery	2.1	1.06–4.85	0.044
Postsurgical cardiogenic shock	2.5	1.28–4.77	0.007
Postsurgical blood transfusion	1.6	1.26–2.78	0.030
Postsurgical pulmonary edema	5.4	2.26–12.84	0.000
Postsurgical pleural effusion	3.0	1.57–5.54	0.001
Retracheal intubation	3.1	1.24–7.51	0.015
Mechanical ventilation time	1.2	1.07–2.31	0.025

OR: odds ratio. CI: confidence interval.

## Data Availability

The original contributions presented in the study are included in the article, further inquiries can be directed to the corresponding author.
